# Prevention of cadmium bioaccumulation by herbal adaptogens

**DOI:** 10.4103/0253-7613.75669

**Published:** 2011-02

**Authors:** K. Bharavi, A. Gopala Reddy, G.S. Rao, P. Ravi Kumar, D. Srinivas Kumar, P. Prabhu Prasadini

**Affiliations:** Department of Pharmacology & Toxicology, NTR College of Veterinary Science, Gannavaram, Krishna (dt), Hyderabad, Andhra Pradesh, India; 1 Department of Pharmacology & Toxicology, College of Veterinary Science, ANGRAU, Rajendranagar, Hyderabad, Andhra Pradesh, India; 2 Department of Animal Nutrition, NTR College of Veterinary Science, Gannavaram, Krishna (dt), Hyderabad, Andhra Pradesh, India; 3 Department of Environmental Science & Technology, College of Agriculture, ANGRAU, Rajendranagar, Hyderabad, Andhra Pradesh, India

**Keywords:** Heavy metal, herbal antioxidants, oxidative stress

## Abstract

**Objectives:**

To evaluate the effect of various herbal adaptogens such as shade-dried powders of *Withania somnifera, Ocimum sanctum, Asperagus recemosus, Andrographis paniculata, Asphaltum panjabinum (Shilajith), Gymnema sylvestre, Spirulina platensis,* and *Panex ginseng* on cadmium (Cd)-induced oxidative stress and its accumulation in broiler chicken.

**Materials and Methods:**

A total of 80 male broiler chicks of day old age were randomly assigned to 10 equal groups. Group 1 birds were fed with basal diet throughout the experiment (1–42 days). Group 2–10 chicks were fed with basal diet containing cadmium at 100 ppm from day 1 to day 28 (4 weeks). From 29^th^ to 42^nd^ day (2 weeks), basal diet alone was fed to group 2 chicks which acted as toxic control and group 3–10 birds were fed with feed containing 0.1% powder of *W. somnifera, O. sanctum, Aspe. recemosus, An. paniculata, Asph. panjabinum (Shilajith), G. sylvestre, S. platensis,* and *P. ginseng,* respectively. Body weight gain, levels of non-enzymatic antioxidants such as reduced glutathione (GSH), lipid peroxidation markers such as thiobarbituric acid reacting substances (TBARS), liver functional markers such as serum alanine transaminase (ALT), kidney functional markers such as blood urea nitrogen (BUN) and serum creatinine and concentration of cadmium in liver and kidney were investigated.

**Results:**

Body weight gains were significantly decreased in birds of groups 2–10 compared to group 1 at the end of 4^th^ week. Supplementation of various medicinal herbs in feed after 4^th^ week significantly improved the body weight gain compared to that in group 2 chicks. The increase in TBARS and decrease in GSH concentrations of liver and kidney tissues in cadmium intoxicated birds were significantly reversed by the above-said herbs. The liver and kidney functional markers were also restored to normal levels. Highest concentration of cadmium was found accumulated in kidney, followed by liver in birds of group 2. Herbal supplementation in groups 3–10 prevented Cd bioaccumulation which was most evident in liver, followed by kidney.

**Conclusions:**

Administration of herbal adaptogens at the rate of 0.1% in feed significantly prevented the bioaccumulation of Cd and reversed the Cd-induced oxidative tissue damage.

## Introduction

In the recent past, extensive mining and indiscriminate industrialization have increased cadmium contamination of environment. Plants readily absorb cadmium from the soil and accumulate it in various parts of the plant.[1] Shellfish such as mussels, scallops and oysters and other fish accumulate cadmium and may become a major source of cadmium exposure for poultry and other livestock fed with fish meal and oyster shell grid as calcium source.[2,3]

Multifractional mechanisms are involved in cadmium-induced reactive oxygen species (ROS) generation. Cadmium inhibits Ca^2+^-ATPase in cell membrane/endoplasmic reticulum and prevents Ca^2+^ export from the cytoplasm. The increased Ca^2+^ then activates the enzymes that generate oxygen free radical (O_2_^−^).[4] Cadmium also participates in Fenton reaction leading to generation of hydroxyl radical (HO^·^).[5] Due to its similar electron configuration, Cd competes with Zn and Cu and affects the dependent enzymes such as Cu–Zn superoxide dismutase (SOD).[6] Due its electron sharing affinity, Cd forms a covalent attachment with sulfhydryl groups of proteins and disrupts the intracellular sulfhydryl homeostasis, which in turn depletes reduced glutathione (GSH).[7]

Low dietary levels of Cd intake by broiler chicken results in its progressive accumulation in various tissues such as liver, kidney, muscle and bone,[8,9] causing oxidative stress which adversely affects the performance and serum biochemical parameters, besides damaging kidney, liver and bursa of fabricius.[10,11]

Cadmium intake at relatively low levels as residues of chicken or other foods, affects the kidney cytochrome P450 dependent monooxygenase system[12] and lowers liver CYP2C8/19 protein content with liver pathology.[13]

Dietary supplementation of ascorbic acid (antioxidant) reverses the lipid peroxidation (LPO) and other adverse effects of Cd on broiler performance[10] and also prevents Cd accumulation in various organs in guinea pigs.[14] Supplementation of Brahma rasayana, a polyherbal preparation, to birds subjected to heat stress, significantly increased the GSH content of liver along with increasing the activities of glutathione peroxidase (GPx) and glutathione reductase (GR) enzymes over control birds. Increased formation of GSH triggers the oxidation reactions mediated by GPx and SOD.[15] The herbs which increase the ability to adapt and avoid damage by environmental factors are called adaptogens. *Withania somnifera, Ocimum sanctum, Asperagus recemosus, Andrographis paniculata, Asphaltum panjabinum (Shilajith), Gymnema sylvestre, Spirulina platensis, and Panex ginseng*^16^,^17^ are potent antioxidants which reduce LPO[18–21] by their potential radical scavenging activity.[21–24] Antioxidants like Spirulina platensis have protective action against organ damage caused by lead.[25]

Currently, the possible toxicity of synthetic antioxidants has been criticized. The interest in natural antioxidants, especially of plant origin, has greatly increased in the recent years.[26] Hence, the present study was conducted to evaluate the effect of herbal adaptogens on Cd bioaccumulation and on scavenging of Cd-induced free radicals.

## Materials and Methods

### 

#### Chicken

A total of 80 male broiler chicks (Cobb strain) of day old age were procured from M/s Venkateswara Hatcheries, Hyderabad, Andhra Pradesh, India, and reared in a battery brooder. These chicks were randomly divided into 10 groups consisting of eight birds in each group. All the birds were provided with respective feed and water ad *libitum* throughout the experiment. Birds of all groups were vaccinated against new castle disease (ND) on 7^th^ and 28^th^ days and infectious bursal disease (IBD) on 10^th^ day. Weekly body weights of all birds were recorded from the day of hatch till the completion of experiment.

#### Experimental design

The study duration was 42 days (6 weeks) and the experiment began at the age of day 1. Cadmium was supplemented to the basal diet as cadmium chloride (SD Fine-Chem Limited, Mumbai, India) at a concentration of 100 ppm level. All herbal adaptogens were mixed to the basal diet at 0.1% level. Basal diet was prepared with standard feed ingredients and its Cd concentration was estimated to be 0.433 ppm. As per National Research Council (NRC), 1980, the maximum tolerable dietary Cd level for bovine, ovine, porcine and avian species is 0.5 ppm.[27] The experimental diets received by various groups of birds are as follows:

Group 1: Basal diet throughout the experiment (1–42 days)
Group 2: Basal diet containing 100 ppm Cd for 28 days (4 weeks), followed by basal diet alone from day 29
Group 3: Basal diet containing 100 ppm Cd till day 28, followed by diet containing root powder of *W. somnifera* at 0.1% level until the end of experiment on day 42
Group 4: Basal diet containing 100 ppm Cd till day 28, followed by diet containing leaf power of *O. sanctum* at 0.1% level until the end of experiment on day 42
Group 5: Basal diet containing 100 ppm Cd till day 28, followed by diet containing root powder of *Aspe. recemosus* at 0.1% level until the end of experiment on day 42
Group 6: Basal diet containing 100 ppm Cd till day 28, followed by diet containing roots and aerial parts powder of *An. paniculata* at 0.1% level until the end of experiment on day 42
Group 7: Basal diet containing 100 ppm Cd till day 28, followed by diet containing air-dried powder of *Asph. panjabinum* (Shilajith) at 0.1% level until the end of experiment on day 42
Group 8: Basal diet containing 100 ppm Cd till day 28, followed by diet containing air-dried leaf powder of *G. sylvestre* at 0.1% level until the end of experiment on day 42
Group 9: Basal diet containing 100 ppm Cd till day 28, followed by diet containing air-dried powder of *S. platenensis* at 0.1% level until the end of experiment on day 42
Group 10: Basal diet containing 100 ppm Cd till day 28, followed by diet containing air-dried root powder of *P. ginseng* at 0.1% level until the end of experiment on day 42

#### Blood and tissue collection form birds

Blood samples were collected from wing veins on 28^th^ and 42^nd^ days from all the birds, without anticoagulant for serum separation. Birds were sacrificed by cervical dislocation on 42^nd^ day and liver, kidney and breast muscle pieces were collected. Excised liver and kidney were thoroughly washed in ice-cold saline (0.9% NaCl). Liver was also perfused with ice-cold saline via portal vein. The saline washed liver and kidney tissue homogenates (10%) were made in ice-cold 0.2 M Tris Hcl buffer (pH 7.2). Finally, the cytosolic sample of liver and kidney homogenates was obtained by centrifuging at 10,000 g for 30 minutes at 4°C.

#### Biochemical analysis

GSH content in liver and kidney was determined by reaction of GSH with 5-5'-dithiobis-2-nitrobenzoic acid (DTNB) as per the method of Moran[28] and its activity was expressed in milligrams of GSH/gram protein. Liver and kidney LPO levels were assessed by determining the amount of thiobarbituric acid reacting substances (TBARS), i.e., malandialdehyde (MDA) produced during the LPO, as per the method of Subramanian[29] and the concentration was expressed in nanomoles of MDA/gram protein.

The activity of serum alanine transaminase (ALT) enzyme (IU/L), the liver function marker, was estimated as described by Bergmeyer,[30] and kidney function markers, serum creatinine (mg/dL) and blood urea nitrogen (BUN) (mg/dL) were estimated as per the methods of Apple *et al*.[31] and Donald *et al*.,[32] respectively. Total protein in liver and kidney homogenates was quantified as per the procedure of Lowry *et al*.,[33] with bovine serum albumin as the standard.

#### Cadmium estimation in tissues

Cadmium concentration (μg/g wet tissue) in samples of liver, kidney and breast muscle was determined with atomic absorption spectrophotometer (NOVAA 300). Dry ashing procedure was used for the metal analysis in organs. Five grams of wet tissue was dried at 100°C for 2 hours. Dried samples were transferred to a cool muffle furnace and the temperature was slowly raised to 450°C and ashed overnight. The ash was dissolved in 2 mL of nitric acid and enough double distilled water was added to make a final volume of 50 mL before the Cd content was determined.[11]

#### Statistical analysis

The values were expressed as mean ± SE and were analyzed using one-way analysis of variance (ANOVA) using Statistical Package for Social Sciences (SPSS) 10 ^th^ version. Differences between means were tested employing Duncan’s multiple comparison test and significance was set at *P* < 0.05.

## Results

### 

#### Body weight gain

The average body weight gain recorded on day 28 of birds in groups 2–10 was found significantly decreased compared to the weight gain in group 1 birds. However, the weight gain recorded on day 42 in groups 3–10, which received herbal adaptogen supplemented feed, was found to be significantly increased compared to the weight gain in group 2 [Table 1].

**Table 1:** Body weight gain (g) of different groups of broiler chicks

*Groups*	*4^th^ week*	*6^th^ week*
1	388.4 ± 5.94	307.3 ± 10.66
2	167.2 ± 9.06	139.4 ± 5.79
3	163.6 ± 11.81	414.4 ± 7.42
4	144.2 ± 8.01	430.9 ± 27.48
5	120.8 ± 8.67	361.1 ± 10.85
6	120.5 ± 13.08	410.9 ± 10.89
7	178.0 ± 13.55	351.3 ± 16.79
8	175.6 ± 9.05	283.0 ± 6.74
9	165.91 ± 7.00	292.5 ± 22.88
10	147.8 ± 10.50	340.8 ± 21.69

Values are expressed as mean ± SE (n = 8); one-way ANOVA (SPSS) was used

#### GSH and tissue LPO levels

**Figure 1:**
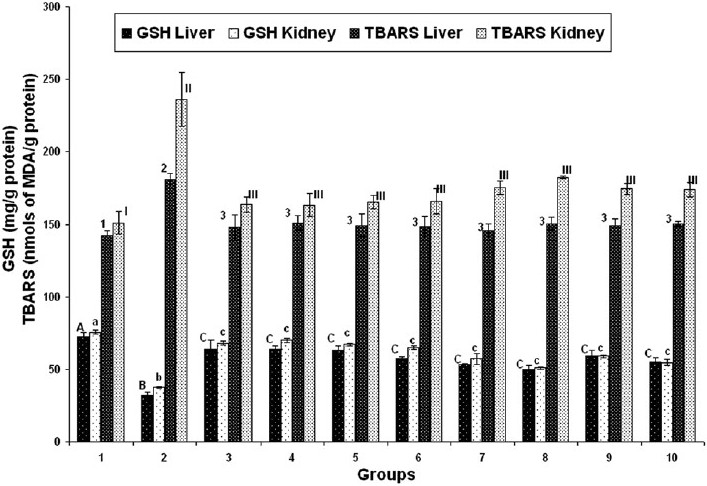
Effect of various herbal adaptogens on cadmium-induced tissue LPO and GSH levels of liver and kidney tissue of broiler chicken (n = 8). Values are expressed as mean ± SE; ANOVA followed by Duncan’s multiple comparison was used. Means with different alphabets as superscripts differ significantly (*P* < 0.05)

Cadmium control group 2 chicks exhibited significant decrease in GSH and increase in TBARS concentration in liver and kidney tissues when compared to those in absolute control group 1. In herbal adaptogen treated groups 3–10, the GSH concentration was found to be significantly increased while the TBARS concentration was found to be significantly decreased in liver and kidney tissues as against those observed in group 2 [Figure 1].

#### Liver and kidney functional markers

**Figure 2:**
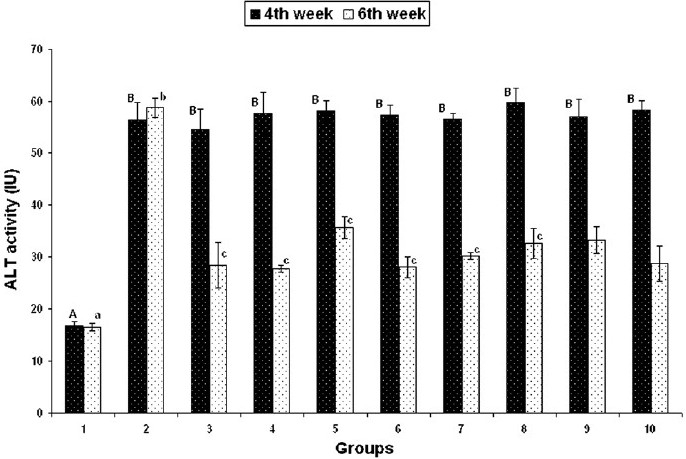
Effect of various herbal adaptogens on cadmium-induced changes in serum ALT activity of broiler chicken (n = 8). Values are expressed as mean ± SE; ANOVA followed by Duncan’s multiple comparison was used. Means with different alphabets as superscripts differ significantly (*P* < 0.05)

**Figure 3:**
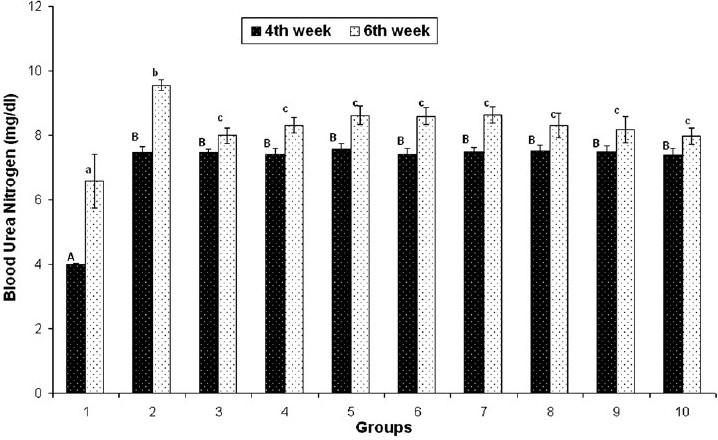
Effect of various herbal adaptogens on cadmium-induced changes in BUN concentration (mg/dL) of broiler chicken (n = 8). Values are expressed as mean ± SE; ANOVA followed by Duncan’s multiple comparison was used. Means with different alphabets as superscripts differ significantly (*P* < 0.05)

**Figure 4:**
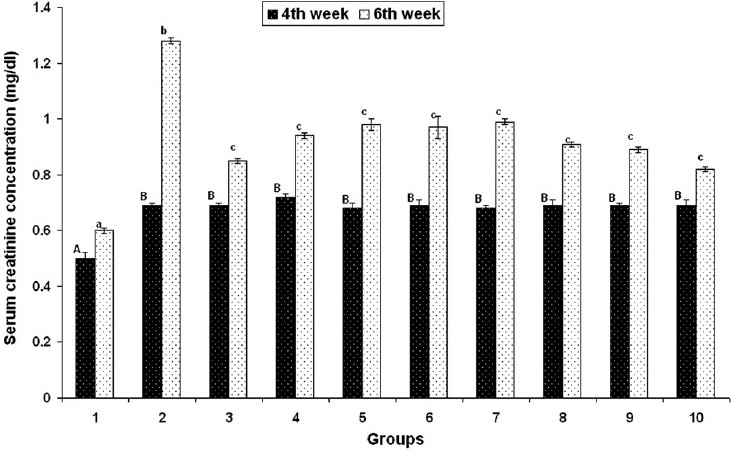
Effect of various herbal adaptogens on cadmium-induced changes in serum creatinine concentration (mg/dL) of broiler chicken (n = 8). Values are expressed as mean ± SE; ANOVA followed by Duncan’s multiple comparison was used. Means with different alphabets as superscripts differ significantly (*P* < 0.05)

On day 28, serum ALT, creatinine and BUN concentrations were found to be significantly increased in groups 2–10, but on day 42 these were found to be significantly decreased in groups 3–10 that received herbal adaptogens, compared to those in group 2 [Figures 2–4].

#### Cadmium concentrations in liver and kidney

Cd accumulation was found in all Cd fed birds (groups 2–10), which was higher in kidney compared to liver. However, in groups 3–10, the Cd bioaccumulation was significantly lowered compared to that in group 2. It was further observed that higher reduction of cadmium accumulation occurred in liver, followed by kidney [Table 2].

**Table 2:** Tissue cadmium levels (µg/g wet tissue) in different groups of broiler chickens

*Groups*	*Cadmium concentration (ppm)*	
	*Liver*	*Kidney*
1.	0.41 ± 0.02	0.38 ± 0.01
2.	67.25 ± 1.87	75.10 ± 2.89
3.	13.28 ± 0.43	41.57 ± 2022
4.	13.74 ± 0.45	41.93 ± 2.91
5.	22.43 ± 1.30	45.93 ± 2.60
6.	20.10 ± 1.09	43.15 ± 2.81
7.	15.15 ± 1.82	42.29 ± 3.21
8.	17.51 ± 1.21	43.81 ± 3.15
9.	15.64 ± 1.08	42.71 ± 2.89
10.	18.19 ± 1.21	44.01 ± 2.54

Values are expressed as mean ± SE; ANOVA followed by Duncan’s multiple comparison was used

## Discussion

Both environmental pollution and scarcity of feed and fodder are increasing day by day. In view of the economics involved, discarding the contaminated feed is not possible always. Hence, means to reduce the toxicity likely to result from consumption of such feeds have to be developed. Antioxidant flavonoids in herbal adaptogens can chelate the catalytic metals and also can act as oxygen free radical scavengers.[34]

In the present study, the cadmium exposure resulted in its significant accumulation in the liver and kidney along with significantly elevated oxygen free radicals and TBARS. Elevated TBARS indicated enhanced tissue peroxidation that led to tissue damage. Significantly increased serum ALT, BUN and creatinine levels reflect the tissue damage thus resulted.

After entering into the cell, the divalent cation cadmium accumulates without apparent toxic effect, predominantly by binding to sulfhydryl groups of protein as cadmium–thionein complex, but when it exceeds the physiological capacity of antioxidant enzymes and GSH, it elevates ROS.[35] In the present study, GSH levels were found to be significantly lowered in cadmium fed birds when compared to that in control. The lowered GSH levels in Cd fed birds can be attributed to the binding of Cd to GSH for its excretion in bile as Cd–GSH complexes and exhaustion of GSH during the process of reduction of HOOH that was elevated by the presence of Cd intracellularly.

**Figure 5:**
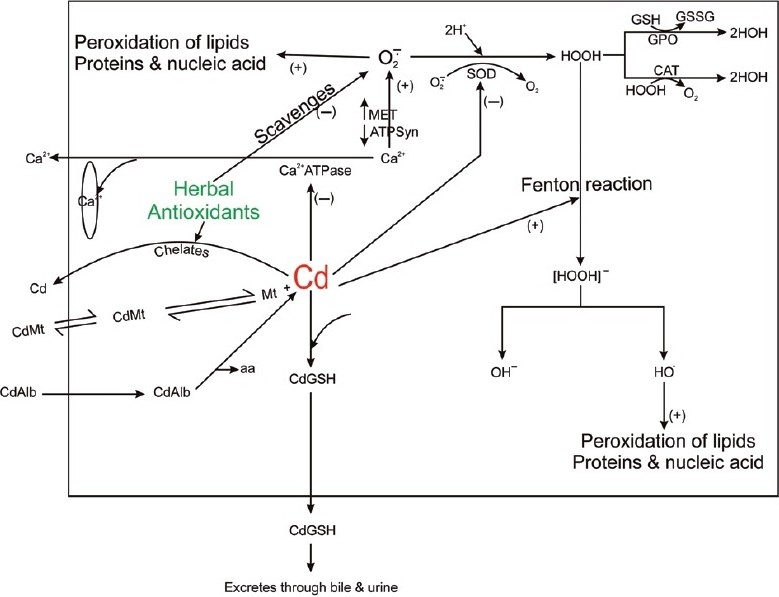
Schematic diagram of mechanism of cadmium-induced free radical generation. Herbal antioxidants scavenge the oxygen free radical and chelate Cd. O¯2: oxygen free radical; HO•: hydroxyl radical; ¯OH: hydroxyl ion; HOOH: hydrogen peroxide; HOH: water; SOD: superoxide dismutase; MET: mitochondrial electron transport; ATPsyn: ATP synthase; aa: amino acid; Cd: cadmium; CdGSH: cadmium– GSH complex; Cdmt: cadmium metallothionine complex; CdAlb: cadmiumalbumin complex; Ca^2^+: calcium ions; CAT: catalase; Ca^2^+-ATPase: calcium ATPase; (+): positive effect; (−): negative effect

Herbal adaptogens such as *W. somnifera, O. sanctum, Aspe. recemosus, An. paniculata, Asph. panjabinum* (Shilajith), *G. sylvestre, S. platensis,* and *P. ginseng* are known for their antioxidant properties. Results of the present study indicate that dietetic supplementation of the above herbs reduced the Cd accumulation in liver and kidney and protected them from subsequent Cd-induced peroxidative damage by free radicals. Herbal antioxidants might have scavenged the oxygen free radicals and averted GSH exhaustion during the process of Cd detoxification. It is further possible that the herbal antioxidants chelated Cd[34] and facilitated its elimination, thereby sparing –SH group of enzymes, GSH and proteins. Elimination of Cd would have restored intracellular Ca ^2+^ to normalcy, which in turn prevented the free radical generation [Figure 5].

It appears from this study that dietetic supplementation of certain antioxidant herbs to Cd intoxicated birds will prevent the Cd accumulation and peroxidative tissue damage.
